# Medical and Philosophical Intelligence

**Published:** 1814-01

**Authors:** 


### MEDICAL AND PHILOSOPHICAL INTELLIGENCE.

### *Account of some Experiments and Observations on Cutaneous Absorption.* By THOMAS SEWALL, M.D.

*EXPERIMENT* 1.—September, 1809. I immersed my feet in an infusion of four ounces of madder in one gallon of water, at the temperature of 99°, that of the atmosphere 65°. The infusion just covered the melleoli interni. In this condition I suffered my feet to remain ten hours. Urine was drawn at the time I placed my feet in the infusion, again at different intervals for thirty hours afterwards.

The first drawn portion was pale and not changed in its colour by adding to it a solution of the vegetable fixed alkali. The portion drawn two hours after was also pale and not changed by the alkaline test. The portion drawn four hours after I placed my feet in the infusion, on the application of the alkali, was evidently reddened. The different portions drawn at the end of the sixth, eighth, tenth, and twelfth hours after I placed my feet in the infusion, were high coloured, and exhibited a bright crimson, on adding to them a few drops of the alkaline test. The bright crimson in the succeeding portions gradually subsided, but the red colour could be distinctly traced in the portion drawn twenty-four hours after I placed my feet in the infusion. Afterwards the red colour was gradually lost. Blood was drawn from the arm just before and after I placed my feet in the bath, but the serum of both portions was of its natural colour, and not changed by the alkali.

*Experiment* 2.—I immersed my feet in an infusion of madder four hours. Urine was drawn when I placed my feet in the infusion, again at the end of the second hour. Neither portions exhibited any change when tested with the alkali. That drawn at the end of the fourth hour was evidently higher coloured, and on adding a few drops of the alkali, instantly assumed a bright red. Urine drawn at the end of the twenty-fourth hour, was of its natural color, and not changed by the alkaline test.

*Experiment* 3.—I immersed my hand and wrist seven hours in an infusion of four ounces of madder in two quarts of water, at the temperature of 98°. Urine was drawn at the time I placed my hand in the infusion, again at the end of the third, fifth, seventh, ninth, eleventh, fifteenth, and twenty-second hours. The first and second portions were of the usual colour, and not changed by the alkali. The portions drawn at the end of the fifth, seventh, and ninth hours, were evidently higher colored, and on adding the alkali, instantly assumed a bright red. The red color in the succeeding portions gradually subsided, and the portion drawn at the end of the twenty-second hour was not altered by the test.

*Experiment* 4.—I immersed my hand and wrist four hours in an infusion of three ounces of rhubarb in two quarts of water, at the temperature of 98°. Urine was drawn at the time I placed my hand in the infusion, and at different intervals for twenty-four hours after. The first portion was of its natural color, and not changed by the alkali. Those portions drawn at the end of the fifth and sixth hours of the experiment, periment, exhibited, on the application of the alkali, an evident change to an orange color.

Since making the above experiments, I have immersed my feet for several hours in infusions and decoctions of redwood, logwood, and oak bark, but have not been able to detect any of their coloring principle in the urine.

---

*Croup.*—In a late case of this terrible disease, a child of fifteen months was bled to the amount of six ounces from the external jugular, *twenty-four hours after the commencement of the disease.* There ensued a very long-continued faintness, and it was manifest that the remedy could not have been employed to a much greater extent, without serious consequences. But the result was happy. In a few hours the respiration altered, and it was nearly natural in twelve hours after the bleeding. A recurrence of the disease the next evening was apprehended, but it did not take place. The child ultimately did well.

---

*Sketches of the State of Medicine in Italy, in* 1812.—At Pavia, the residence of the celebrated M. Scarpa, is an admirable institution. The medical professors are ten in number; the professorships are thirteen. They are as follow: the institutes of surgery; clinical medicine; botany; clinical surgery, by Scarpa; human anatomy, by Fattori; operative surgery, by Scarpa; pathology and legal medicine; chemistry, by Brugnatelli; materia medica; physiology and comparative anatomy; pharmaceutic chemistry, by Marabelli; midwifery.—In addition to these are professors in the following departments: agriculture; experimental philosophy, by Volta and Configliachi; natural history; general physics.

An extensive library belongs to the school, and a splendid museum of anatomy and the various branches of natural history. These are contained in a building much like the medical school in Paris, but twice as large. The hospital is an excellent one, and under remarkably good management. The industry and superior advantages of the head of the institution, M. Scarpa, have contributed greatly to the benefit and improvement of the school. Scarpa was the pupil of Morgagni, and colleague with Fontana. At the early age of eighteen he was professor of anatomy at Modena. After this he was a pupil of William Hunter in London, and then came to settle in Pavia. He has recently finished an improved gorget for the operation for the stone, one advantage of which is that it enters the bladder with very little force on the part of the operator. M. Scarpa will also publish an instrument for performing lithotomy above the pubis.

M. Brugnatelli, the professor of chemistry, is making a very large collection of concretions found in the animal body. To facilitate his object he has sent circular letters to the medical men in Italy, soliciting specimens of this nature. These applications have been very successful, if one may judge from the extent of his cabinet. He finds that in some of his specimens, the different layers of calculus are composed of essentially different principles. One of his specimens shews a great analogy between the animal and vegetable systems. He has a piece of rhubarb in which is imbedded a calcareous concretion in every every respect resembling those found in animals, and appearing to have been gradually formed there.

M. Brugnatelli edites a large periodical work, a great part of which is taken up by the reports of his own labours.

Pisa is the seat of a medical school to which is attached a considerable hospital. But the establishment may with more propriety be said to belong to Florence, for the professors generally reside in that city and hold their examinations there. To Fontana's cabinet of anatomical preparations in wax at Florence, is annexed a small collection of preserved animals and skeletons in comparative anatomy, also a very extensive collection of minerals and marine productions, and a hortus siccus well displayed. Within the grounds belonging to the establishment is a botanical garden, possessing every convenience for the preservation of tender plants. The physical apparatus belonging to the academy is pretty extensive. The two globes of copper and lead with which the famous experiment of compression was made are preserved here, also the telescope with which Galileo made some of his most important discoveries.

Florence has two extensive and well established hospitals; one civil, the other military. The latter contains an unusual number of lunatics, many of whom have become so from the effects of the conscription, or of sudden and extreme poverty.

---

Mr. Tardy, surgeon, late of Marchmont-street, has just established a house for the reception of insane persons. The numbers received at this institution being very limited, Mr. Tardy trusts that he can accommodate patients in a manner superior to what can be expected in establishments upon a larger scale, as well in regard to the internal arrangements of the house, as to the facility which will be afforded of exercise out of doors, by the appropriation of between two and three acres of pleasure and garden grounds, the whole secured from external observation, being surrounded by several acres of meadow land, belonging to the same proprietor, and situated in a very airy and healthy situation, about ten miles from London.

Mr. Tardy, meaning to confine his practice in future to the treatment of insane persons, and with his family on the spot, will be able to secure to those who are received into his asylum, both male and female patients, every attention and assistance which the general source of their disease on any emergency may require. Communications addressed to Mr. Tardy, surgeon, at his office, 14, Marchmont-street, Brunswick-square, will be immediately attended to.

---

*New Operations for Cataract.*—An experiment of the most important kind has recently been tried upon the pensioners of Greenwich Hospital, by direction of the Honorable Governors of that Institution, with a view to ascertain the comparative success of different operations for cataract. The operation of extraction had been performed, it appears, upon the blind pensioners, for the last fifteen or twenty years, by the celebrated oculists Mr. Wathen, and by his successor Mr. Phipps, but not, it is understood, with very satisfactory termina-

 tions.

tions. The Governors have now appointed Mr. Adams to be oculist to the Hospital, (where all the blind men in the navy are sent when invalided;) that gentleman has performed a series of novel operations for cataract, upon a large number of patients, with singular success. We have not been informed of the peculiarities in Mr. Adams's newest operations, nor have accurate intelligence of the comparative results of the *old* and *new* methods; but those results, we have learned, are decidedly in favor of the latter.

---

*Note from* Mr. P. Lee *to the Editor, on the Effects of Ammonia in preventing Contagion.*

Sir,

It is now more than twenty-six years since I first used it, under the impression that contagion might be in itself an acid sui generis, or combined with some acid which had not been ascertained by analysis: taking this for granted, I thought the ammoniacal vapor once in contact would neutralize the principle, and so far decompose the sceptic matter of contagion as to render it inert, or tertium quid. For this purpose I made use of the liquor ammoniæ, by frequently sprinkling the bed and room where I was in attendance on typhus patients, and for some time observed that not one of the visitants or attendants caught the fever. I shall detail the case of one family in proof of this circumstance:—John Wright, pilot, of South Shields, in the county of Durham, in the year 1800, himself, wife, and five children, were all in a state of typhus fever; the man, his wife, and one child, were in bed, two upon chairs, and two on the floor, all cooped up in one small room, the weather at that time being very sultry. Prior to my being called in, a married woman and a girl of thirteen years of age caught the fever; and although their relatives and friends visited them after the ammoniacal vapor was used, not one was affected. Lately, when the alarm was given that the plague was in Wapping, I went in quest of it, with a full determination of being barricaded in with whom I could find affected, hoping I should find the ammoniacal vapor as efficacious in preventing the contagion of plague as it did of typhus, by which I should arrest the disease on its first appearance; and being well aware that one method I use in stopping mortification would prevent the carbuncles degenerating into a state of gangrene, I should have entered into it with full confidence of success, well assured government would have supplied me with every requisite. The nitrous fumigation of Dr. Carmichael Smith may be equally efficacious; but when it is considered that congestion in the lungs frequently attends typhus fever, any excitement producing cough must increase it. The ammoniacal vapors do not produce this effect; and if a sufficient quantity of liquor ammonia was allowed to prisons, ships, and other places liable to low nervous fevers, it would be the means of preventing a disease which is the pest of this country.

38, *Rathbone Place, Oxford Street,* P. Lee.
*Dec.* 29, 1813.

The DEATHS within the Bills of Mortality this Year are—

| | |
|---|---|
| Abortive & Stilborn | 630 |
| Abscess | 57 |
| Aged | 1571 |
| Ague | 2 |
| Apoplexy and Suddenly | 292 |
| Asthma | 574 |
| Bedridden | 5 |
| Bleeding | 30 |
| Bursten & Rupture | 19 |
| Cancer | 83 |
| Canker | 1 |
| Childbed | 186 |
| Colds | 13 |
| Colic, Gripes, &c. | 8 |
| Consumption | 4736 |
| Convulsions | 3239 |
| Cough & Hooping Cough | 389 |
| Cow Pox | 1 |
| Cramp | 2 |
| Croup | 85 |
| Diabetes | 3 |
| Dropsy | 698 |
| Evil | 4 |
| Fevers of all kinds | 714 |
| Fistula | 6 |
| Flux | 7 |
| French Pox | 11 |
| Gout | 34 |
| Gravel, Stone, Strangury | 11 |
| Grief | 5 |
| Headmoldshot, Horshoehead, and Water in the Head | 297 |
| Inflammation | 741 |
| Inoculation | 2 |
| Itch | 1 |
| Jaundice | 34 |
| Jaw Locked | 2 |
| Leprosy | 1 |
| Lethargy | 1 |
| Livergrown | 45 |
| Lunatic | 207 |
| Measles | 550 |
| Mortification | 205 |
| Palpitation of the Heart | 6 |
| Palsy | 144 |
| Pleurisy | 19 |
| Piles | 1 |
| Quinsy | 5 |
| Rash | 1 |
| Rheumatism | 8 |
| Scurvy | 3 |
| Small Pox | 898 |
| Sore Throat | 4 |
| Sores and Ulcers | 13 |
| Spasm | 24 |
| St. Anthony's Fire | 4 |
| Stoppage in the Stomach | 25 |
| Surfeit | 2 |
| St. Vitus's Dance | 2 |
| Swine Pox | 1 |
| Teeth | 288 |
| Thrush | 44 |
| Tumor | 2 |
| Water in the Chest | 27 |
| Worms | 1 |

CASUALTIES:

| | |
|---|---|
| By the Explosion of Gunpowder | 1 |
| Bit by a mad Dog | 1 |
| Broken Limbs | 1 |
| Burnt | 35 |
| Drowned | 101 |
| Excessive Drinking | 4 |
| Executed | 12 |
| Found Dead | 9 |
| Frighted | 4 |
| Killed by Falls & several other Accidents | 80 |
| Killed themselves | 35 |
| Murdered | 4 |
| Poisoned | 3 |
| Scalded | 3 |
| Suffocated | 5 |

| | | | |
|---|---|---|---|
| Christened | Males | 10608 | In all 20528 |
| | Females | 9920 | |
| Buried | Males | 8993 | In all 17322 |
| | Females | 8329 | |

Whereof have died,

| | |
|---|---|
| Under Two Years of Age | 5167 |
| Between Two and Five | 1733 |
| Five and Ten | 604 |
| Ten and Twenty | 526 |
| Twenty and Thirty | 1108 |
| Thirty and Forty | 1501 |
| Forty and Fifty | 1751 |
| Fifty and Sixty | 1506 |
| Sixty and Seventy | 1559 |
| Seventy and Eighty | 1211 |
| Eighty and Ninety | 489 |
| Ninety and a Hundred | 61 |
| A Hundred | 1 |
| A Hundred and One | 1 |
| A Hundred and Two | 1 |
| A Hundred and Nine | 2 |
| A Hundred and Thirteen | 1 |

Decreased in the Burials this year, 973.

On

On the important subject of definite proportions, Professor Berzelius, of Stockholm, draws the following conclusions as laws of formation:

1. In a chemical combination of two or more oxidated bodies, (whether it consists of acid and acid, of acid and base, or of base with base,) the oxygen of the substance which is most abundant, is a multiple by a whole number (1, 2, 3, 4, . . .) of the oxygen of the body which is least abundant; and in every chemical combination between two combustible substances, they are present in such quantities, that, if the compound be oxygenized, a new combination will be formed, which follows the same law.

2. In organized productions, two, three, or more inflammable substances are united, and attached to a single portion of oxygen, which is only sufficient for the oxygenization of one of them; and this combination cannot be divided into more immediate component parts, nor be formed from such parts.

---

Dr. Richard Powell will, in January, commence a Course of Lectures on the Practice of Medicine, at St. Bartholomew's Hospital.

Dr. Clement Hill will commence his Course of Lectures on General and Pharmaceutical Chemistry, on Wednesday, January 23d, at the same Hospital.

Mr. Abernethy will commence his Spring Course of Lectures on Natural and Morbid Anatomy, and Physiology, on Thursday the 20th of January, at two o'clock, at the above Hospital.

---

*Theatre of Anatomy, Bartlett's-court, Holborn.*—Mr. John Taunton, F.A.S. Member of the Royal College of Surgeons of London, Surgeon to the City and Finsbury Dispensaries, City of London Truss Society, &c. will commence his Winter Course of Lectures on Anatomy, Physiology, Pathology, and Surgery, on Saturday, January the 22d, 1814, at eight o'clock in the evening precisely, and to be continued every Tuesday, Thursday, and Saturday, at the same hour.

In this Coruse of Lectures it is proposed to take a comprehensive view of the structure and economy of the living body, and to consider the causes, symptoms, nature, and treatment of surgical diseases, with the mode of performing the different surgical operations; forming a complete course of anatomical and physiological instruction for the medical or surgical student, the artist, the professional or private gentleman.

An ample field for professional edification will be afforded by the opportunity which pupils may have of attending the clinical and other practice of both the City and Finsbury Dispensaries.

---

*Theatre of Anatomy, Blenheim-street, Great Marlborough-street.*—The Spring Course of Lectures on Anatomy, Physiology, and Surgery, will be commenced on Monday, the 24th of January, at two o'clock, by Mr. Brookes.

Anatomical Converzationes will be held weekly, when the different subjects treated of will be discussed familiarly, and the student's views forwarded. To these none but pupils can be admitted.

Spacious

Spacious apartments, thoroughly ventilated, and replete with every convenience, are open all the morning for the purposes of dissecting and injecting, where Mr. Brookes attends to direct the students, and demonstrate the various parts as they appear on dissection.

---

Dr. Squire will, on Saturday, January the 8th, begin a Course of Lectures on the Theory and Practice of Midwifery, and the Diseases of Women and Children, at his house, in Ely-place, Holborn.

---

Dr. Clough, Physician-Accoucheur to the St. Mary-le-bone General Dispensary and Endeavour Society, will commence his Winter Course of Lectures on the Science and Practice of Midwifery, including the Diseases of Women and Infants, as connected therewith, about the middle of January 1814, at his house, 68, Berners-street. Morning course at half-past ten; evening course at seven.—Students, when properly qualified, will have the same extensive opportunity of attending cases as usual, for practical improvement, in regular progression; and their views forwarded by weekly examinations.

---

Dr. Clarke and Mr. Clarke will begin their third Course of Lectures on Midwifery and the Diseases of Women and Children, on Monday, January 24th, 1814. The Lectures are read at the house of Mr. Clarke, No. 10, Upper John-street, Golden-square, every morning from a quarter past ten to a quarter past eleven, for the convenience of students attending the hospitals.

---

Dr. Merriman, Physician-Man-Midwife to the Middlesex Hospital, the Westminster General Dispensary, and the Parochial Infirmary of St. George, Hanover-square, will recommence his Course of Lectures on Monday, January 24th, at half-past ten o'clock, at the Middlesex Hospital.

---

Mr. Singer will commence a Course of Lectures on Experimental Philosophy, on Tuesday the 18th of January. The Lectures will be continued on the succeeding Tuesdays and Fridays.

---

Mr. Stevenson, Surgeon-Oculist and Aurist to her Royal Highness the Princess of Wales, Member of the Royal College of Surgeons, &c. will recommence his annual Course of Lectures on the Anatomy, Physiology, and Diseases of the Eye and Ear, including their medical and surgical treatment, on the 25th of January.—The introductory Lecture will be delivered by Mr. Stevenson, at his house, 105, Great Russel-street, Bloomsbury-square, precisely at eight in the evening, to which medical practitioners and students may, as usual, have free admission.

